# Effects of Training for First Milking Involving Positive Tactile Stimulation on Post-Calving Maternal Behaviors in Primiparous Gyr Dairy Cows

**DOI:** 10.3390/ani13050921

**Published:** 2023-03-03

**Authors:** Rogério Ribeiro Vicentini, Lenira El Faro, Aska Ujita, Maria Camila Ceballos, João Alberto Negrão, Aline Cristina Sant’Anna

**Affiliations:** 1 Programa de Pós-Graduação em Biodiversidade e Conservação da Natureza, Universidade Federal de Juiz de Fora (UFJF), Juiz de Fora 36036-900, MG, Brazil; 2 Centro Avançado de Pesquisa de Bovinos de Corte, Instituto de Zootecnia (IZ)–Agência Paulista de Tecnologia dos Agronegócios/Secretaria de Agricultura e Abastecimento (APTA/SAA), Sertãozinho 14160-970, SP, Brazil; 3 Faculty of Veterinary Medicine, University of Calgary, Calgary, AB T3R 1J3, Canada; 4 Faculdade de Zootecnia e Engenharia de Alimentos (FZEA), Departamento de Ciências Básicas, Universidade de São Paulo (USP), Pirassununga 13635-900, SP, Brazil; 5 Núcleo de Estudos em Etologia e Bem-estar Animal (NEBEA), Departamento de Zoologia, Universidade Federal de Juiz de Fora (UFJF), CNPq Researcher, Juiz de Fora 36036-900, MG, Brazil

**Keywords:** aggressiveness, cow–calf contact systems, maternal protective behavior, maternal care, Zebu

## Abstract

**Simple Summary:**

Maternal care and protective behavior have important roles in offspring survival. For Zebu dairy cattle, these maternal behaviors are even more relevant, influencing both productive performance and handler safety, as many dairy farms use cow–calf contact systems. The objectives of this study were to investigate the effects of a training protocol involving pre-calving positive tactile stimulation on the post-calving maternal care and protective behavior of primiparous Gyr cows. These cows (n = 37) were allocated into two groups: training (*n* = 16) and control (*n* = 21). Various maternal behaviors were evaluated. Calf latency to stand up, body weight, and sex influenced cow–calf interactions, whereas the training group cows touched less and spent more time not interacting with their calves. Both the training and control groups had protective dams, but a higher percentage of the trained group dams were calmer toward calf handling. We concluded that the primiparous dairy Gyr cows subjected to the pre-calving positive tactile stimulation training protocol tended to be less protective on the first handling of their calves and presented less maternal care.

**Abstract:**

In dairy systems with Zebu breeds, calves are not immediately separated from their dams after calving; consequently, maternal care and protective behavior are important, influencing both productive performance and stockpeople’s safety. Our objectives were to: (1) investigate the effects of a training protocol involving pre-calving positive stimulation, delivered prior to calving, on the maternal care of primiparous Gyr cows; and (2) evaluate the effects of this training protocol on maternal protective behavior towards handlers during the first calf handling. Primiparous dairy Gyr cows (*n* = 37) were allocated into two groups: training (*n* = 16) and control (*n* = 21). Animal behaviors were recorded in three periods: post-calving, first calf handling, and post-handling. Maternal protective behavior during calf handling was assessed from measures of aggressiveness, attention, displacement, and agitation. Calf latency to stand up (*p* < 0.01) and sex (*p* < 0.01) differed between the training and control groups. The training group had less touching (*p* = 0.03), more time not interacting with the calf (*p* = 0.03), tended to be less protective (*p* = 0.056), and moved less (*p* < 0.01) during the first handling of their calves. In conclusion, the primiparous dairy Gyr cows subjected to pre-calving training protocol displayed less maternal care and displacement during the first handling of their calves and tended to be less protective.

## 1. Introduction

Improving human–animal interactions addresses worldwide societal demands for better animal welfare, an impetus for improvement in diverse animal production fields involving animal handling [1,2]. The quality of human–animal interaction affects the welfare of several dairy species, e.g., cattle, buffaloes, sheep, and goats (reviewed by Napolitano et al. [3]). On dairy farms, the importance of improving human–animal relationships is evident as animals are handled on a daily basis for milking and have direct contact with handlers [4,5].

There are various ways to improve animal handling and consequently animal welfare, including a selection of handlers [6], improving facilities [7,8], or gentle handling procedures [9,10,11]. Gentle handling, e.g., stroking body regions, gentle tactile stimulation, and vocal interactions in European dairy cattle (*Bos taurus taurus*) improved animal responses to humans and handling routines [10]. Stroking the body regions of Brown Swiss and Austrian Simmental lactating cows, Schmied et al. [12] reported that gentle stimulation reduced avoidance distance and increased approaches to humans. Lürzel et al. [10] reported that gentle tactile stimulation and vocal interactions with Holstein–Friesian heifers induced behavioral responses compatible with pleasure during stroking. In Gyr (*Bos taurus indicus*) dairy cattle, pre-calving brushing stimulation reduced cows’ reactivity score, respiratory rate and rectal temperature [13]. Similarly, primiparous and multiparous Gyr cattle subjected to positive tactile stimulation in the prepartum period reduced reactivity during milking, e.g., stepping and kicking [14]. The training protocol was more efficient in primiparous versus multiparous cattle to maintain adequate behaviors and milk ejection during their first milking [14]. Paranhos da Costa et al. [15] reported beneficial effects of a training protocol on Hostein–Gyr dairy heifers reducing reactivity during first milking and facilitating the milking routine. These results demonstrated strategies to address management challenges during milking. However, for dairy cows, the most challenging handling procedures may start immediately after calving.

On tropical dairy farms, heifers are usually infrequently handled and maintained in pasture-based systems before first calving and when handling procedures occur, they are generally aversive or neutral (e.g., vaccination, hot-iron branding, artificial insemination, weighing). These less-frequent and generally aversive interactions can cause greater fear and consequently reactive responses to human presence and handling procedures [16,17]. It can be aggravated in the peripartum period, which is very sensitive for both dam and calf, further complicating management. In dairy farms with European breeds, calves are usually separated from their dams within the first 24 h of life [18,19]. However, on most pasture-based farms with Zebu dairy breeds, the separation of dam and calf is not performed, as it may compromise lactation persistence [20,21]. Thus, the first handling of dams and calves after calving can be challenging for Zebu dairy cattle. Handling newborn calves is necessary for healthcare, including navel asepsis, weighing, and identification [22]; however, these procedures can be hampered by the dam’s presence [23]. Cows that perceive these interventions as a potential threat to their calf may exhibit extreme protective behaviors leading to a high risk of accidents to stockpeople and calves [24,25]. Issues related to behavior and the handling of recently calved cows are relevant in the livestock industry as aggressive cows can compromise one-welfare and farm sustainability [23,24,26,27].

In this context, positive handling could be used in day-to-day farm management to improve the welfare and safety of animals and handlers [9,10,11,14]. Perhaps a previous positive experience (e.g., habituation to humans and gentle handling) can mitigate the aggressive reactions of recently calved cows toward humans [23]. Additionally, Flörcke et al. [28] speculated that calmer cows would display mothering problems and perhaps reduced maternal behaviors toward offspring, but they did not provide supporting data. Furthermore, there was a low phenotypic correlation but a moderate genetic correlation between docility and maternal behavior (measured by time licking the calf) [29]. Thus, it is plausible to expect a possible association between cow calmness and maternal performance. However, to our knowledge, no studies have evaluated effects of the gentle tactile stimulation protocols before calving on maternal behavior. The effects of a training protocol on the protective behavior of Zebu dairy cows in cow–calf contact systems are also unknown.

Thus, using primiparous Gyr dairy cows, we wanted to determine the effects of a training protocol for milking, involving gentle tactile stimulation prior to calving, on: (1) maternal care during the post-calving period; and (2) maternal protective behavior towards handlers during the first handling of calves. We hypothesized that: (1) the training protocol would affect the cows’ maternal behavior; and (2) cows subjected to the training protocol would have lower protective behavior against stockpeople during the first handling of their calves.

## 2. Materials and Methods

### 2.1. Animals and Handling

Thirty-seven primiparous Gyr dairy cows (*Bos taurus indicus*), aged 46.26 ± 8.63 months, from an experimental farm station (Empresa de Pesquisa Agropecuária de Minas Gerais-Epamig Oeste, Uberaba, Minas Gerais State, Brazil) were used. Only eutocic calvings of singleton births between August and December 2017 were included. A pasture-based system was used for all cattle. Cows were allocated into two groups: training (*n* = 16) and control (*n* = 21), based on the expected calving date (the first cow to calve was randomly allocated to one group, the next cow to calve was then allocated into the alternate group, with this pattern continuing for the remaining cows). In the training group, 40 days before the estimated calving day, cows started a training protocol for the first milking, involving gentle tactile stimulation (14 days consecutively) in a conventional tandem parlor (12 milking machines, in two rows). The protocol was performed by six trained handlers and constituted three phases, as described by Ujita et al. [14]. Briefly, in Phase 1, cows were driven from the pasture to the milking parlor and passed through the milking stalls; in Phase 2, cows went through the milking stalls and were brushed (2 min) on the whole body (head, neck, trunk, udder, front legs, and hind legs); and in Phase 3, cows went through the milking stalls, their bodies (mainly udder and hind legs) were brushed (2 min), legs restrained and teat asepsis (pre-dipping) was performed. Additional information about the milking parlor and milking routine is described in Ujita et al. [14]. The control group did not receive the training protocol and was subjected to typical farm management. At 30 days before expected calving, the cows of both groups were relocated from the pasture to a maternity paddock (0.55 ha; maximum stocking density: 27 cows/hectare), with both the control and training group cows kept in the same paddock. The nutritional management of both groups in the maternity consisted of corn silage and 500 g concentrate per cow, delivered to the feeder twice a day using a tractor vehicle. Additionally, water and mineral salt were offered ad libitum. Cows weighed 425.5 ± 47.3 kg (training group: 427.5 kg ± 37.1 kg; control group: 422.5 ± 37.1 kg). Details about the maternity paddock and nutritional management of cows are fully described in Vicentini et al. [30]. During the final gestation period, cows were not handled or disturbed (except for the training group during the protocol). All routine procedures (i.e., feed delivery, calf weighing, and navel asepsis) were performed by the same six handlers trained in good practices of cattle handling.

### 2.2. Behavioral Observations

The maternity paddock was equipped with four video monitoring cameras (GIGA, GSHDP20TB) that recorded cow behavior 24 h a day. Cows were individually identified with non-toxic paint (Koleston, Wella^®^, Darmstadt, Germany) on both sides of their body. Calving was defined as complete expulsion of the calf. Thereafter, a minimum of 3 h were allowed for the cow–calf dyad to remain together without any human intervention, with the first calf’s handling performed afterward. Calf handling was performed daily during handlers’ working hours (8 am–5 pm); therefore, cows that calved from 5 pm to 4 am remained with their calves for 3 to 15 h.

Three periods were considered to record the maternal behaviors: (1) ‘post-calving period’: 3 h following the complete expulsion of the calf; (2) ‘first calf handling’: the period of calf handling, including inspection and navel asepsis; (3) ‘post-handling period’: 1 h after completion of navel asepsis. After the ‘post-handling period’, both cow and calf were removed from the maternity pen. Based on video recordings from monitoring cameras, 189 h (4.5 h/cow) were analyzed. A single trained observer recorded the cow behaviors using focal sampling and continuous observation [31]. During the ‘post-calving’ period, the latency of the first calf touch by the cow (‘cow latency’), and the latency of the calf to stand on its four feet (‘calf latency’) were recorded in minutes (Table 1). Unsuccessful attempts by calves to stand until they were able to stand up without falling (‘calf attempts’) were recorded as number of occurrences (Table 1). Behaviors related to cow–calf interaction (‘touching’, ‘not interacting’ and ‘suckling’) in both the ‘post-calving’ and ‘post-handling’ periods were recorded as percentages of observation time (Table 1).

During the ‘first handling’ period, cow protective maternal behavior was assessed by a single trained observer using two scoring systems, ‘maternal composite score (MCS)’ and ‘maternal protective behavior (MPB)’, as described by Vicentini et al. [32]. The MCS was obtained by adding the scores of the four main behaviors: ‘aggressiveness’ (1–3), ‘attention’ (1–3), ‘displacement’ (1–5), and ‘agitation’ (1–4) [33]. Full descriptions of the scores are included as the Supplementary Material (Table S1). The sums of the MCS scores ranged from 4 (minimum) to 10 (maximum) and subsequently transformed from 1 into 7. The MPB applied scoring from 1 (calm cows) to 5 (aggressive cows) [32]. Navel disinfection was conducted by two familiar handlers that worked in pairs. The handlers’ approach was standardized, aiming to be consistent and avoid influencing the cows’ behavior, as described by Vicentini et al. [32]. After the ‘post-handling period’, the cow and calf were moved from the maternity paddock to the corral where the colostrum milking and calf identification procedures occurred and the calf was weighed (Digitron Scale).

### 2.3. Statistical Analyses

Descriptive statistics and normality tests were conducted for all behavioral variables using the PROC Univariate of Statistical Analysis System (SAS^®^ Institute, INC., Cary, NC, USA). To evaluate the effects of the treatment (training group vs. control group) on cow and calf behaviors, and maternal protection scores, general linear models were fitted, using PROC GLIMMIX of SAS and adopting the lognormal distribution for variables with non-normal distribution (‘cow latency’, ‘calf latency’, ‘calf attempts’, MCS, and MPB). Cows’ behaviors (‘cow latency’, ‘touching’, ‘not interacting’, and ‘suckling’) and calf behaviors (‘calf latency’ and ‘calf attempts’) at ‘post-calving’ and ‘post-handling’ periods, and maternal protection scores (MCS and MPB) were used as dependent variables. Treatment (training group vs. control group), ‘calf sex’ (male or female), treatment, and calf sex interaction were used as the fixed effects. The ‘Calf weight’ (in kilograms) and cow age (in months) were included as covariates with linear effects. 

Complementarily, Chi-square tests in contingency tables were used to estimate the associations between treatment (training or control groups) with scores for aggressiveness, attention, displacement, and agitation. Pearson correlation coefficients were used to investigate the relationships between cow and calf behaviors in ‘Post-calving’ and ‘Post-handling’ periods. *p* values ≤ 0.05 were considered significant and *p* values ≤ 0.10 tendencies.

## 3. Results

There were more male (*n* = 20) than female (*n* = 17) calves, and males tended to be heavier (Table 2). There were effects of ‘calf sex’ (F = 9.94; *p* < 0.01) and ‘calf sex’ * ‘calf weight’ interaction (F = 6.97; *p* = 0.01) on ‘calf attempts’ to stand up; male calves had almost double the number of attempts than females (Table 2). 

Regarding cow behaviors, the ‘calf weight’ (F = 3.33; *p* = 0.07) tended to affect the ‘cow latency’, with heavier calves taking longer to be touched by their dams. There were relationships between the cow and calf behaviors. A positive correlation between the ‘calf latency’ and ‘calf attempts’ (r = 0.63; *p* < 0.01) indicated that the calves with the longest time to stand up were also those that made more attempts to do so. In addition, a positive correlation between ‘calf latency’ with ‘not interacting’ in both the ‘post-calving’ (r = 0.36; *p* = 0.03) and ‘post-handling’ (r = 0.41; *p* = 0.01) periods was detected. Finally, a tendency between ‘calf attempts’ and ‘touching’ (r = −0.30; *p* = 0.09), and a significant correlation between ‘touching’ and ‘not interacting’ (r = −0.94; *p* < 0.01), both at ‘post-calving period’ were identified.

Regarding the effects of a training protocol on maternal behaviors in the ‘post-calving period’ there was a significant effect of the treatment * ‘calf sex’ interaction on the variables ‘touching’ (F = 4.79; *p* = 0.03) and ‘not interacting’ (F = 4.85; *p* = 0.03). Male calves were less touched than females by cows from the training group, but there was no difference in the control group (Table 2). The training group dams of male calves spent less time ‘touching’ and more time ‘not interacting’ than those from control group (Table 2). During the ‘post-handling period’, there was an effect of the ‘calf weight’ on the ‘touching’ behavior (F = 3.96; *p* = 0.05), in which heavier calves were touched longer by their dams. Cows from the training and control groups did not differ in other assessed behaviors.

**Figure 1:**
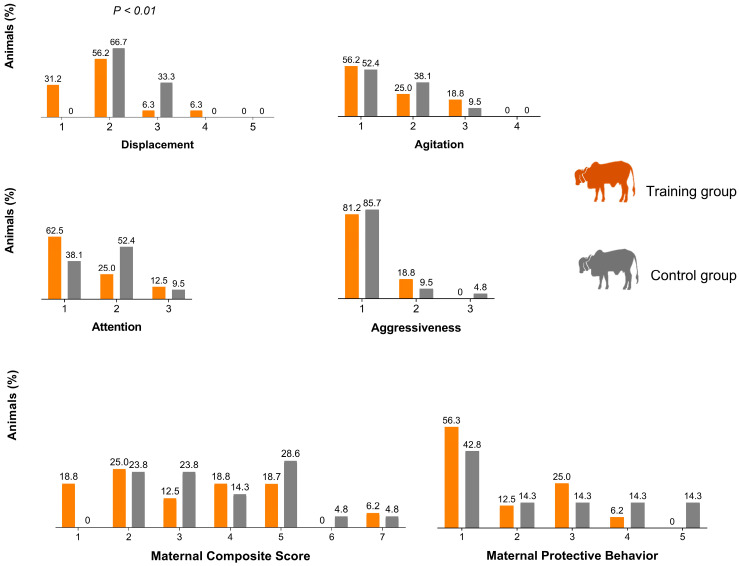
Distribution of displacement, agitation, attention, and aggressiveness scores, maternal composite score (MCS), and maternal protective behavior (MPB) of primiparous Gyr dairy cows at the first handling of their calves.

Distributions of the scores for ‘displacement’, ‘agitation’, ‘attention’ and ‘aggressiveness’ plus ‘maternal composite score’ (MCS), and ‘maternal protective behavior’ (MPB) are presented in Figure 1. Treatment group had a tendency on MCS (F = 3.92; *p* = 0.056; trained: 3.18 ± 1.75 vs. control: 3.80 ± 1.47) and an effect on ‘displacement’ (F = 10.05; *p* < 0.01; trained: 1.87 ± 0.80 vs. control: 2.33 ± 0.48) scores. Scores for ‘agitation’, ‘attention’, ‘aggressiveness’ and MPB did not statistically differ between treatment groups. Chi-square revealed an association between treatment with ‘displacement’ score, with higher percentage of score 0 for the training group and a higher percentage of score 3 for the control group (χ^²^ = 11.11; *p* < 0.01). For all other measures, there were no significant associations with treatment groups (*p* > 0.05).

## 4. Discussion

Maternal behavior is an important trait in many domestic species [34,35]. However, in Zebu breeds, this characteristic is even more relevant as it impacts both dam productivity and calf performance [25,36]. In the present study, male calves tended to be heavier and made more attempts to stand up. Despite being heavier, the higher frequency of attempts combined with the higher latency of males to stand for the first time can be understood as a signal of lower vigor compared to female calves. Lower motility can be a reliable indicator of low vigor in Zebu calves [37]. Indeed, at birth, male calves have greater chance of poor vigor than females [38,39]. Calf vigor must be considered an important indicator of offspring survival. In Nellore cattle, Schmidek et al. [40] reported the risks of low vigor and death were ~20% greater in males compared to females.

Several factors influence the postpartum behavior of cows, with an important role of calf vigor in arousing the mother’s interest [41,42]. In our study, heavier calves experienced a delay in being touched by their dams. One possible explanation was exhaustion from the calving process. Although dystocia did not occur in our study, cows delivering heavier calves can experience severe pain or exhaustion during calving [43]. This phenomenon was evinced by correlations between cows and calf behaviors (longer latency to stand up was related to less maternal touch and interactions). Edwards and Broom [41] reported the influence of weariness on delayed standing after calving, which can also affect first maternal care (e.g., touching, cleaning, and suckling). Additionally, pain signs and weariness can be more evident in primiparous than multiparous cows [32,44], and our studied cows were all primiparous. Future studies could include calving duration as a potential indicator identifying cows with more strenuous efforts to deliver heavier calves, resulting in longer intervals of rest to recover before approaching the calf.

In addition to vigor, calf sex appears to have an important role in triggering cows’ nursing behavior. Female calves were touched and interacted more with their dams than males during the post-calving period. This behavior can be attributed to the exhaustion from the calving process. As discussed above, male calves tended to be heavier, likely resulting in a more exhausting calving process. Detrimental effects of difficult calving (e.g., pain and fatigue have been reported and may impair maternal care [41,43,45]). Stěhulová et al. [46] studying Gasconne cattle reported that cows provided more maternal care to low-weight calves, and licked and followed more female than male calves. Similarly to taurine cattle, Zebu cows of the Guzerat breed were described as spending more time and providing more maternal care to low-weight calves [47].

Training influenced the maternal care delivered by cows with male calves, with the training group cows spending less time performing maternal care behaviors (less ‘Touching’ and more ‘Not interacting’) than cows in the control group. This was the first evidence of a possible effect of a training protocol to the first milking and/or use of gentle tactile stimulation on maternal behavior in Zebu cattle, since no previous studies have evaluated this topic. Further studies are needed to better elucidate biological reasons for the effects of training only being manifested in the dams of male calves. Previous studies reported reductions in fear, reactivity, and aversiveness during the first milking in response to gentle tactile stimulation for Gyr and Holstein–Gyr cows [13,14,15]. Additionally, Flörcke et al. [28] speculated that very calm cows would have limited maternal performance. Unfortunately, in the present study, we did not perform a temperament assessment of these cows in the absence of their calves to confirm whether trained cows were calmer or more docile than control cows during handling. We can only presume that the gentle handling and training protocols may modulate some aspects of maternal behavior towards their calves, that can be dependent on calf sex.

Regarding maternal protectiveness, control group cows were more displaced during the first handling of their calves and tended to be scored as more protective based on MCS, without significant effects on the MPB, aggressiveness, attention, or agitation scores. Lower maternal defense responses toward humans in trained cows were expected, based on previous evidence that habituation and gentle interactions decrease aversive and avoidance reactions toward people in cattle, and improve human–animal interactions [10,11,48]. Evaluating beef Gyr, Brahman, and Gyr–Brahman cross cows, Orihuela et al. [23] observed that more aggressive cows towards handlers were those with more intensive protective behavior reaction to separation from their calves during handling. However, the authors did not report any relationship between the aggressive behaviors and cow temperament in the peripartum period [23]. Beef cows are recurrently described to be more protective of their calves than dairy cows [27,35,49]. Using qualitative behavior assessment, Ceballos et al. [33] reported that dairy Holstein–Gyr cows with aggressive behavior during their calves handling were those considered more ‘frightened’ and ‘irritated’ than the cows classified as ‘loving’ and ‘attentive’. Although, in general, Zebu dams demonstrate strong protective behavior to their calves, we emphasize the plasticity of this phenotype, influenced by several factors [25,50]. In this case, environmental effects and handling routine may have modulated maternal behavior. Repeated handling and habituation are useful tools to improve animal responses to handling [50,51]. Cows with lower milking reactivity, better productivity, and calmer response when restrained were already described as a result of positive tactile stimulation protocol [13,14]. Despite having a weak effect on maternal protectiveness, we attributed the reduced displacement and tendency of a lower MCS to gentle tactile stimulation received during training. Habituation to human presence and gentle handling may influence how cattle perceive humans [23]; cattle may regard them as something other than a potential risk for their calves.

Although the effects documented herein suggest a relationship between the training protocol and maternal performance, our study had some limitations, requiring thoughtfulness to extrapolate our results. Even though we observed a tendency of the training protocol to affect maternal protectiveness (MCS score) of Gyr dairy cows, the differences between individuals were important. Some aspects of these individual traits may influence a stimuli response even in standardized protocols [15]. Individuals’ past experiences (before conception or even during gestation) may have masked or influenced cow behavior in some way, mainly toward handlers [23,48]. Furthermore, we only had 16 primiparous cows in the training group and a single group per treatment (without replication). Therefore, pre-milking training effects on maternal protectiveness must be confirmed in future studies with greater sample sizes and preferably using a replicated design with both male and female offspring. Moreover, it is important to evaluate intra-observer reliability when measuring animal behavior as it gives confidence about the quality of assessment made by the same observer; unfortunately, we did not evaluate it in our study. Nevertheless, the observer has been trained in observing animal behavior with years of experience in this task. Future studies should consider including a baseline (pre-calving) reactivity assessment of both groups (trained and control) to better elucidate the potential effects of the training protocol on cows’ reactivity post-calving. Finally, more studies should investigate the possible effects of training protocols for longer intervals after calving, evaluating the long-term effects on cow–calf bonds across lactation in cow–calf contact production systems.

## 5. Conclusions

A pre-calving training protocol for the first milking involving gentle tactile stimulation on primiparous Gyr dairy cows was associated with reduced displacement and lower maternal defense, based on a maternal composite score, which can be beneficial for the handling routine of calves. However, primiparous Gyr dairy cows subjected to the training protocol spent less time touching their male calves. In addition, maternal care was also affected by calf latency to stand up, weight, and sex. The potential long-term effects of lower maternal care by trained cows on calf development need to be determined in future studies. In general, our findings bring new perspectives about using a training protocol to habituate primiparous cows to the first milking. In addition to the relevant benefits already known in productivity and animal welfare, the adoption of a training protocol could modulate the protective responses of dams to the management of their calves.

## Figures and Tables

**Table 1:** Ethogram of primiparous Gyr dairy cow and calf behaviors in Post-calving and Post-handling periods.

Categories	Description
*Post-calving period*	
**Cow latency** (min ^a^)	Period from complete expulsion of the fetus (calving) to the first time the cow touches the calf with the muzzle and/or tongue.
**Calf latency** (min.)	Period from expulsion of the fetus (calving) to the calf stands on four legs without falling.
**Calf attempts** (freq ^b^)	Number of calf unsuccessful attempts to stand.
*Post-calving and Post-handling periods*	
**Touching** (% ^c^)	Cow’s tongue or muzzle is in physical contact with any part of the calf’s body.
**Not interacting** (%)	Cow is standing or lying without physical contact and/or without any type of physical interaction with the calf.
**Suckling** (%)	Cow is standing still while the calf sucks her teats, or makes contact with teats and/or udder region.

^a^ min = latency in minutes; ^b^ freq. = frequency (in number); ^c^ % = percentage of observation time.

**Table 2:** Means (±standard error) of primiparous Gyr dairy cow behaviors and calf sex, behavior, and weight during the post-calving and post-handling periods.

Categories	Mean ± SEM	Treatments	
		Training	Control
**Calf sex** (freq ^1^)			
Male _(♂)_		11	9
Female _(♀)_		6	11
**Calf weight** (kg ^2^)	22.70 ± 0.52	23.62 ± 0.91	22.05 ± 0.61
Male _(♂)_	23.29 ± 0.72 ^a^*	23.72 ± 1.22	22.92 ± 0.87
Female _(♀)_	21.87 ± 0.73 ^b^*	23.42 ± 1.43 ^A^*	21.02 ± 0.76 ^B^*
**Calf latency** (min ^3^)	62.51 ± 7.51	68.87 ± 11.16	57.66 ± 10.25
Male _(♂)_	68.28 ± 11.18	73.80 ± 15.77	63.27 ± 16.38
Female _(♀)_	54.93 ± 9.38	60.66 ± 15.13	51.50 ± 12.44
**Calf attempts** (freq.)	13.54 ± 1.00	14.06 ± 1.47	13.14 ± 1.38
Male _(♂)_	16.28 ± 1.38 ^a^**	15.80 ± 1.93	16.72 ± 2.05
Female _(♀)_	9.93 ± 0.82 ^b^**	11.16 ± 1.83	9.20 ± 0.72
	*‘Post-calving Period’*		
**Cow latency** (min.)	4.60 ± 0.81	4.75 ± 1.54	4.50 ± 0.91
Male _(♂)_	5.30 ± 1.24	5.40 ± 2.21	5.23 ± 1.49
Female _(♀)_	3.64 ± 0.89	3.66 ± 2.01	3.63 ± 0.92
**Touching** (% ^4^)	52.95 ± 2.81	49.38 ± 4.48	55.72 ± 3.56
Male _(♂)_	48.92 ± 18.36	42.96 ± 4.87 ^B,b^**	55.63 ± 7.32 ^A^**
Female _(♀)_	57.51 ± 11.59	60.95 ± 6.77 ^a^**	55.80 ± 3.11
**Not interacting** (%)	42.00 ± 2.87	45.63 ± 4.66	39.17 ± 3.57
Male _(♂)_	47.43 ± 4.49	53.12 ± 5.07 ^a,A^**	41.03 ± 7.33 ^B^**
Female _(♀)_	35.85 ± 2.81	32.15 ± 5.91 ^b^**	37.69 ± 3.08
**Suckling** (%)	3.94 ± 0.90	4.04 ± 1.20	3.85 ± 1.32
Male _(♂)_	2.90 ± 3.78	3.39 ± 1.57	2.36 ± 0.90
Female _(♀)_	5.11 ± 6.18	5.22 ± 1.95	5.05 ± 2.25
	*‘Post-handling Period’*		
**Touching** (%)	38.91 ± 2.99	36.87 ± 4.10	40.53 ± 4.33
Male _(♂)_	39.73 ± 4.36	38.00 ± 5.76	41.66 ± 6.91
Female _(♀)_	37.98 ± 4.18	35.00 ± 5.80	39.61 ± 5.77
**Not interacting** (%)	56.41 ± 3.09	56.45 ± 4.27	56.37 ± 4.50
Male _(♂)_	53.50 ± 4.53	54.00 ± 6.25	52.96 ± 6.98
Female _(♀)_	59.66 ± 4.16	60.55 ± 4.84	59.17 ± 6.03
**Suckling** (%)	3.47 ± 0.94	3.95 ± 1.46	3.08 ± 1.26
Male _(♂)_	4.47 ± 1.40	3.66 ± 1.69	5.37 ± 2.35
Female _(♀)_	2.35 ± 1.23	4.44 ± 2.90	1.21 ± 1.15

^1^ freq. = frequency (in number); ^2^ kg: kilograms; ^3^ min = latency in minutes; ^4^ % = relative frequency; ^a–b^ In the same column of each category, means without a common lowercase superscript indicate significance (*p* ≤ 0.05) ** or tendency (*p* ≤ 0.10) *; ^A–B^ In the same row of each category, means without a common uppercase superscript indicate significance (*p* ≤ 0.05) or tendency (*p* ≤ 0.10).

## Data Availability

The data presented in this study are available on request from the corresponding author.

## Supplementary Materials

The following supporting information can be downloaded at: https://www.mdpi.com/article/10.3390/ani13050921/s1, Table S1. Scores used to assess maternal protectiveness, including the maternal protective score (MPS), displacement (DIS), agitation (AGI), attention (ATT), and aggressiveness (AGG) scores as described by Ceballos et al.
