# Supplementary material for: Effects of Training for First Milking Involving Positive Tactile Stimulation on Post-Calving Maternal Behaviors in Primiparous Gyr Dairy Cows
Source: Animals (Basel). 2023 Mar 3;13(5):921. doi: 10.3390/ani13050921 (PMC10000114; doi:10.3390/ani13050921)
Supplement: Supplementary file 1 [file animals-13-00921-s001.zip › animals-2128148-supplementary.pdf]

Supplementary Table S1. Scores used to assess maternal protectiveness, including the maternal protective score (MPS), displacement (DIS), agitation (AGI), attention (ATT), and aggressiveness (AGG) scores as described by Ceballos *et al.*[33].

Traits	Scores	Descriptions
**MPS**	1	The cow is distant or moves away from the calf at the time the handler approaches, remaining distant during handling but looking back at the calf sometimes.
	2	The cow is distant or away from the calf when the handler approaches, remaining distant during handling, but keeps its gaze directed towards the calf more than half of the observation time, and may eventually approach and/or lick and/or sniff the calf quickly.
	3	The cow is close to the calf and does not walk away, or walks away and returns quickly at the time the handler approaches, remaining close to the calf more than half of the observation time and keeping her gaze always on him, she may smell and/or lick the calf and/or the handler for a long time.
	4	The cow does not walk away from the calf when the handler approaches; she exhibits threatening behaviors (gaze directed towards the handler and/or head up or may show continuous head movement and/or displacement towards the handler, who is on foot), but does not attack him. In this case, another handler approaches the cow on a horseback, moving her away from the calf, allowing the handler to perform the calf handling.
	5	The cow does not walk away from the calf, threatens and attacks the handler, not allowing him to approach on foot. In this case, the second handler (on a horseback) is also threatened (continuous head movement and/or displacement toward the mounted handler), or attacked by the cow.
**DIS**	1	The cow remains still for all the observation time.
	2	The cow remains still for more than half of the observation time, walks for less than half of the observation time.
	3	The cow walks more than half of the observation time;
	4	The cow walks all the observation time.
	5	The cow moves all the observation time, trotting or galloping.
**AGI**	1	The cow does not present any sudden movement of the head and/or ears and/or tail during all observation time.
	2	The cow does not present sudden movements of the head and/or ears and/or tail in more than half of the observation time.
	3	The cow presents sudden movements of the head and/or ears, and/or tail for more than half of the observation.
	4	The cow presents sudden and frequent movements of the head, and/or ears, and /or tail, during all observation time.
**ATT**	1	The cow is with eyes, and/or ears directed to the handler and/or her calf during the entire observation time.
	2	The cow is with eyes, and/or ears directed towards the handler and/or her calf for more than half of the observation time.
	3	The cow is the eyes, and/or ears directed opposite side from the handler and/or her calf for more than half of the observation time.
**AGG**	1	The cow does not threaten the handler during observation time.
	2	The cow threatens the handler when he approaches the calf but does not attack.
	3	The cow threatens the handler when mounted on a horse and/or attacks the handler (whether mounted on horseback or not).
